# A Learning Health System Framework to Operationalize Health Data to Improve Quality Care: An Australian Perspective

**DOI:** 10.3389/fmed.2021.730021

**Published:** 2021-10-27

**Authors:** Joanne C. Enticott, Angela Melder, Alison Johnson, Angela Jones, Tim Shaw, Wendy Keech, Jim Buttery, Helena Teede

**Affiliations:** ^1^Southern Synergy, Department of Psychiatry, Monash University, Melbourne, VIC, Australia; ^2^Monash Centre for Health Research and Implementation, Monash University, Clayton, VIC, Australia; ^3^Monash Partners Academic Health Science Centre, Clayton, VIC, Australia; ^4^Sydney Health Partners, Sydney, NSW, Australia; ^5^Faculty of Medicine and Health, University of Sydney, Sydney, NSW, Australia; ^6^Health Translation South Australia, Adelaide, SA, Australia; ^7^Centre for Health Analytics, Department of Paediatrics, University of Melbourne, Parkville, VIC, Australia

**Keywords:** data-driven learning, learning health care system, healthcare improvement, quality, translational

## Abstract

Our healthcare system faces a burgeoning aging population, rising complexity, and escalating costs. Around 10% of healthcare is harmful, and evidence is slow to implement. Innovation to deliver quality and sustainable health systems is vital, and the methods are challenging. The aim of this study is to describe the process and present a perspective on a coproduced Learning Health System framework. The development of the Framework was led by publicly funded, collaborative, Academic Health Research Translation Centres, with a mandate to integrate research into healthcare to deliver impact. The focus of the framework is “learning together for better health,” with coproduction involving leadership by an expert panel, a systematic review, qualitative research, a stakeholder workshop, and iterative online feedback. The coproduced framework incorporates evidence from stakeholders, from research, from data (practice to data and data to new knowledge), and from implementation, to take new knowledge to practice. This continuous learning approach aims to deliver evidence-based healthcare improvement and is currently being implemented and evaluated.

## Introduction

Data and benchmarking alone do not drive healthcare improvement, and core challenges remain, with estimates of around 30% of care is low value and 10% potentially harmful (1). Furthermore, effective sustainable healthcare improvement appears to be an intractable problem. There is a recognized vital need for systems-level change to improve healthcare using an iterative learning health system (LHS) approach (1, 2). The LHS broadly encompasses the operationalization and conversion of routinely collected health data into useful information to enable informed, timely decisions to improve quality healthcare and health outcomes (2–8). Herein, we describe the coproduction process and the outcome for the development of the Monash Partners LHS framework, integrating research and data utilization into healthcare to improve outcomes.

This framework development occurred in the context of publically funded Academic Health Science Centres (AHSC) in Australia, where AHSCs have a strong focus on research translation (9, 10) and are tasked with driving “Better Health through Research.” Monash Partners Academic Health Science Centre (Monash Partners) is a partnership between leading health services, teaching, and research organizations, accredited by the Australian National Health and Medical Research Council (NHMRC). The mission of Monash Partners is to connect researchers, clinicians, and the community to innovate for better health and deliver health impact. Monash Partners led this work engaging all 10 NHMRC accredited Australian Research Translation Centres, under the auspices of the Australian Health Research Alliance (AHRA: www.ahra.org.au). AHRA has a national reach across 95% of academic and research teams of Australia, and over 80% of acute health services are collaborating to improve health nationally (9). AHRA formed a data-driven healthcare improvement national system level initiative to “improve health outcomes across our community, through data-driven innovation and care.” In this context, Monash Partners led a rigorous national priority setting process across AHRA centres, communities, healthcare, government, and other stakeholders. The top priority was to create data-driven hubs for healthcare improvement or LHS, across the AHRA centres.

With ever increasing data availability, there is a growing interest in how best to use it to inform decision making in healthcare delivery (2). Systems are needed to ensure the most relevant information, and evidence can guide healthcare decision making (11, 12). Improved healthcare requires systems in which routine data, from service delivery, and patient care, can lead to iterative cycles of knowledge generation and improvement in healthcare, as a result of daily practice (5, 6). Informed decision making is needed at all levels of healthcare, including decisions made by policymakers, hospital executives, clinicians, and by patients themselves (13). In this perspective article, we outline the codevelopment process, present the codesigned framework, and describe the ongoing coproduction of the LHS as it is implemented, evaluated, and scaled through government funding.

### Evidence Synthesis and Codesigning a LHS

The LHS was developed using a multistep codesign process including; engaging the national data-driven healthcare improvement committee across the centres and establishing leadership through the Monash Partners data governance committee with consumer and stakeholder input; obtaining resources through the Australian Government Medical Research Future Fund; and appointing a fellow (JE) and jointly agreeing on a vision and undertaking a rigorous process to develop the framework. We synthesized evidence on systematic review and qualitative research and completed workshops and consultations. The framework was codesigned with stakeholders, with coproduction in implementation and scale-up. Our stakeholders played an integral role throughout from foundational design, ongoing development, current implementation, embedding, and operationalizing the framework evaluating measurable health care improvement.

National governance was established through the data-driven healthcare improvement committee, and the initial priority setting process occurred with nominated members from each competitively accredited Research Translation Centre, consumers, and stakeholder representatives (9). Detailed methods and results of the systematic review and qualitative research are published elsewhere (7, 8) and summarized here.

### Collective Vision and Evidence Collection

The codesign process involved multidisciplinary stakeholders including community, clinicians, academics, administrators, and industry and generated a collective vision of “Learning together for better health” to guide framework development.

### Systematic Literature Review

The systematic review captured the academic and gray literature evidence on effective LHS (or similar entities with alternative names) that stimulated partnerships across multiple stakeholders and increased the translation of data and research in healthcare, with explicit evidence of health impact (8).

Forty-three articles were identified, which described research translation leading to impact in 23 LHS environments: United States (*n* = 18), Canada (*n* = 2), and one each in the UK, Sweden, and Australia/New Zealand. Key findings are summarized in Box 1 and the full systematic review is published (8).

Box 1 **Key findings of the LHS systematic review.**

• Learning Health System environments are system level initiatives with effective examples demonstrating taking practice to data, integrating best practice evidence, undertaking data analysis to generate new knowledge, and implementing new knowledge back into clinical practice in an ongoing, systems level approach

• An integrated multidisciplinary team of frontline clinicians, researchers, and community members, embedded in healthcare settings is key to success

• To have direct health impact, a Learning Health System must provide timely access to data, as well as analysis of that data with feedback

• Effective Learning Health Systems require people with a broad range of workforce capacities to make sense of the data arising from complex healthcare environments

### Qualitative Interviews

The expert panel and systematic review had informed the questions explored in the qualitative research. We purposively identified and conducted semistructured qualitative interviews with national and international leaders, including in the UK and Canada, experienced in supporting or developing data-driven innovations in healthcare (7). Representatives from all AHRA centres, Monash Partners member organizations, the Digital Health Collaborative Research Centre, State Government, Australian Digital Health Agency, Public Health Research Network, consumers and international experts from both the UK and Canada, were interviewed. Analysis of 26 interviews revealed five themes, integral to an effective, sustainable LHS, as shown in Box 2. Full details of the qualitative research are published (7).

Box 2 **Key themes that emerged from qualitative interviews on a learning health system.**

• Structure, governance, trust, culture, vision, and leadership were all seen as important along with a skilled workforce and sustained investment

• Broad stakeholder, clinician and academic engagement, with collective vision, leadership, governance and a culture of trust, transparency, and co-design

• Resourcing with sustained investment over time

• Skilled workforce, capability, and capacity building

• Data access, systems, and processes

• Systematic approaches and iterative, continuous learning with implementation into healthcare contributing to new best-practice care to improve outcomes

### Stakeholder Workshop

The expert panel and systematic review had informed the qualitative research, and learnings from these were integrated into a draft high-level framework and principles. This was followed by iterative stakeholder engagement *via* the members on the governance committee from partner organizations and finally within a stakeholder consultation workshop to refine the proposed model, ensuring adherence to the vision and alignment with end-user needs. The stakeholder consultation workshop was of 4 h duration and involved 60 representatives from Monash Partners organizations, government, national data agencies, AHRA centres, and consumers. It was facilitated by an experienced consultative facilitator. The workshop presented background and project findings, presentations by the state government chief information officer, and by academic clinicians who provided examples of effective LHS. Three of the authors (JE, HT, and AJ) presented the evidence gathered from the systematic review and qualitative interviews, as well as the related priorities established with these partner organizations in earlier related work (9). Immediately after a presentation on the draft LHS framework and principles, participants were divided into groups of ~10 people per group and asked to provide input to refine the proposed draft framework. Each group workshopped at least one quadrant of the LHS framework with instructions to provide input to refine the model elements to improve alignment to the vision and end-user requirements. At the end of this session, a spokesperson from each group presented their inputs and suggestions for the LHS framework, and the facilitator supported the wider group to ask questions and make additional comments and/or suggestions for improvement. Written workshopped papers were collected by the researchers at the end of the workshop and transcribed into a report. Immediately after the workshop, two of the authors (JE and AJ) documented their key impressions arising from the discussions in the workshop by the participants and later incorporated this into the report. The feedback was incorporated into the LHS framework and sent out to participants electronically for comments, and further electronically iteratively refined to generate the final framework.

### Monash Partners LHS

**Figure 1:**
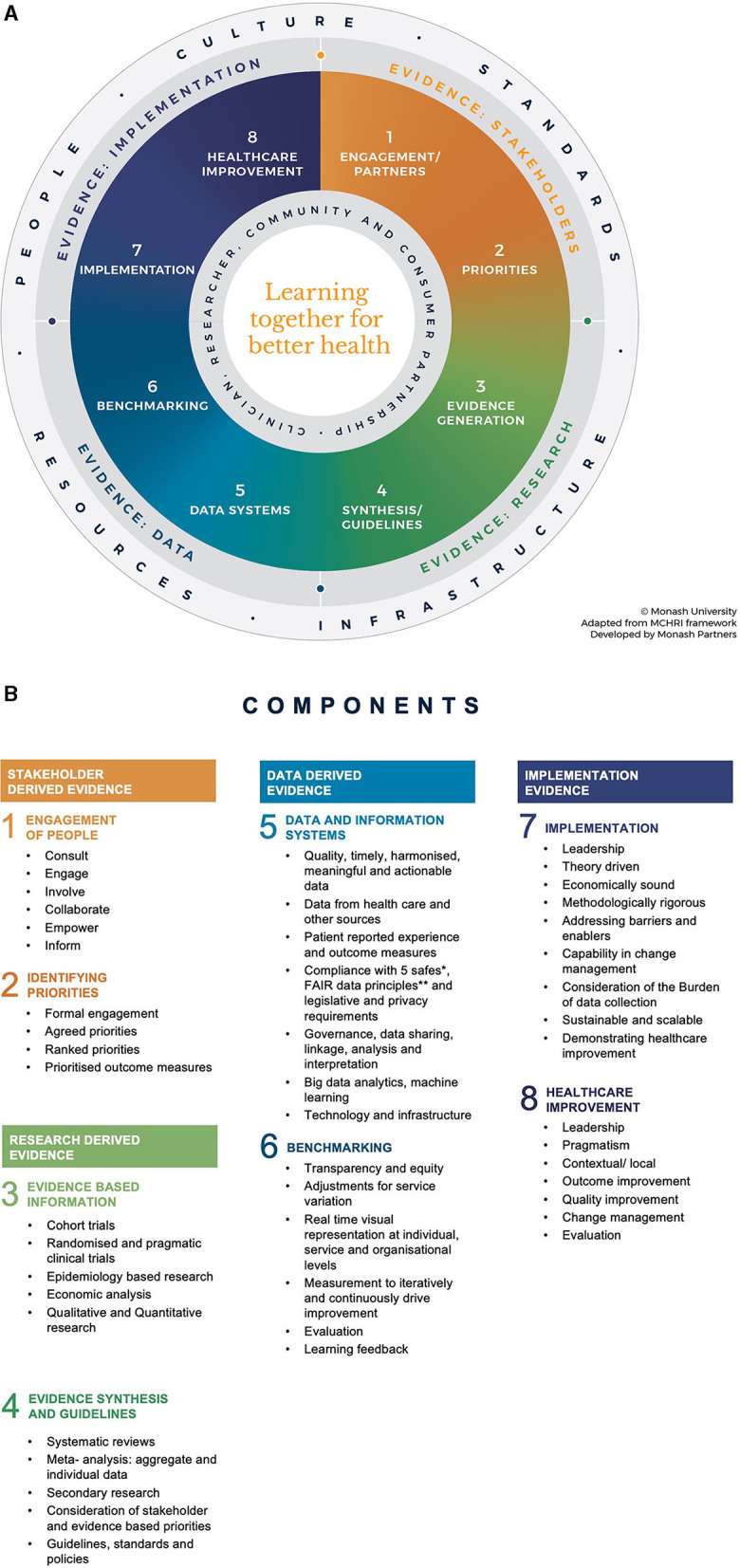
**(A)** Monash Partners Learning Health System framework. The framework shows four key sources of evidence, with each represented diagrammatically in a quadrant of the Learning Health System cycle. **(B)** Bullet points list the topics and functions incorporated under each sub-section in the overarching LHS framework. The numbers correspond to the numbered sections in the LHS framework shown in **(A)**.

The final framework (Figure 1) encapsulates core phases across stakeholder-engagement and priority setting, integration of evidenced based best practice, taking routine health practice data from service delivery and patient care, analyzing this to generate new knowledge, and implementing this new knowledge back into practice in iterative cycles of data-driven healthcare improvement.

The framework is in the shape of a “circle” divided into four main quadrants (Figure 1A). Topics and functions for each quadrant are listed (Figure 1B). The framework shape and contents were synthesized using evidence from the systematic review, qualitative research, and consultation workshop.

The framework shows four key sources of evidence, with each represented diagrammatically in a quadrant of the LHS cycle (see Figure 1):

- Evidence of the stakeholder—from end user problems and priorities
- Research evidence—from primary research, evidence synthesis, and guidelines
- Data evidence—from practice data and data analysis, including artificial intelligence
- Implementation evidence—integrating rigorous implementation research into pragmatic healthcare improvement.

Each quadrant of evidence is vital to capture, identify, and address health service and community priorities and emergent challenges and needs to be integrated to create and operationalize the LHS as an iterative systems level intervention to deliver health impact.

## Discussion and Implementation Activity

Our healthcare system faces a burgeoning aging population, rising complexity, rapid advances in technology, and escalating costs. Around 10% of healthcare is harmful, evidence is slow to implement, and system reform is challenging (1). Innovation to deliver quality and sustainable health system is vital, and methods are controversial and challenging. Here, we describe the codevelopment process of the framework to guide health care settings into becoming LHS. We present a rigorously developed LHS Framework grounded in NHMRC accredited Research Translation Centres (which are publically funded academic health science centres) with a mandate to integrate research into healthcare to deliver impact. The coproduced framework takes practice to data, data to new knowledge, and new knowledge to practice in a continuous learning cycle, to deliver evidence-based healthcare improvement and is currently being implemented and evaluated.

Whilst there are multiple different frameworks in use, most are derived from singular perspectives, be that a single health condition or an isolated research or healthcare perspective, and few consider the consumer and stakeholders as key to the system (2–6, 11–13). Given that current healthcare improvement strategies and conventional project-based approaches to transform care have been inadequate (1), a systems-level approach is required for sustainability and scalability. However, it is important that the LHS is broad and considers all dimensions of the complex adaptive system to succeed.

Frameworks for LHS have been described (2–6, 11–13), and each follows a similar cycle of assembling, analyzing, and interpreting data, followed by feeding the learnings back into practice and creating changes (2). We used this evidence-based process in Australia to develop the LHS framework through stakeholder engagement, and systematic review of LHSs that have delivered impact, qualitative interviews, and workshops contain the key components to succeed. Key components that emerged were evidence sources coming from stakeholders, data, implementation evaluation, and research.

There is clear support from both State and Federal Government health departments, for the LHS, with financial support for a number of projects. The Victorian state government has invested in the LHS in the Victorian healthcare recovery initiative to improve care delivery as we emerge from the COVID-19 pandemic (14). The processes involved engaging community, clinical networks, state government, and health service priorities including new evidence-based models of Telehealth and virtual care and reducing low-value care. Best practice evidence was sourced in these fields, including the Digital Health Cooperative Research Centre resources and the Choosing Wisely and Evolve low-value care initiatives (15). Practice data are being sourced and analyses and implementation are underway. This work is being evaluated at a project and LHS level. State Government funding is supporting data integration systems, and a process led by Monash Partners and a grant through the Medical Research Future Fund is supporting the development of data infrastructure within the LHS: “Towards a National Data Management Platform supporting Australia's Learning Health System.” This initiative will utilize the LHS to support the implementation of a consistent approach to Data Sharing Agreements and Principles, modification, and utilization of systems that will support access to electronic medical records' unstructured data, across a number of health settings and will also link into interstate LHS initiatives through the AHRA network.

Monash Partners is now working across other Centres, partner organizations, Government, and stakeholders, and is funded to implement the LHS frameworks and pilot healthcare improvement projects to iteratively “learn together for better health.”

## Data Availability Statement

The original contributions presented in the study are included in the article/cited material, further inquiries can be directed to the corresponding author/s.

## Ethics Statement

This program of work was approved by the Monash University Human Research and Ethics Committee (Project ID: 19969).

## Author Contributions

HT, AJoh, AJon, and JE lead and participated in the project initiative at all key stages. JE and AJoh participated in data collection. AJoh, AJon, and HT facilitated the workshop. JE drafted the paper with guidance from AJoh, AM, AJon, and HT. All authors reviewed the draft manuscript, provided critical feedback with recommendations, reviewed and approved the final manuscript, conceptualized and designed the study, and participated in data analysis.

## Funding

This work was supported by a fellowship program for one of the author (JE) from Monash Partners and funded by the Medical Research Future Fund.

## Conflict of Interest

The authors declare that the research was conducted in the absence of any commercial or financial relationships that could be construed as a potential conflict of interest.

## Publisher's Note

All claims expressed in this article are solely those of the authors and do not necessarily represent those of their affiliated organizations, or those of the publisher, the editors and the reviewers. Any product that may be evaluated in this article, or claim that may be made by its manufacturer, is not guaranteed or endorsed by the publisher.
